# *Illicium verum* (Star Anise) and *Trans*-Anethole as Valuable Raw Materials for Medicinal and Cosmetic Applications

**DOI:** 10.3390/molecules27030650

**Published:** 2022-01-19

**Authors:** Marta Sharafan, Karolina Jafernik, Halina Ekiert, Paweł Kubica, Ryszard Kocjan, Eliza Blicharska, Agnieszka Szopa

**Affiliations:** 1 Chair and Department of Pharmaceutical Botany, Medical College, Jagiellonian University, ul. Medyczna 9, 30-688 Kraków, Poland; marta.sharafan@gmail.com (M.S.); karolina.jafernik@doctoral.uj.edu.pl (K.J.); halina.ekiert@uj.edu.pl (H.E.); p.kubica@uj.edu.pl (P.K.); 2 Department of Analytical Chemistry, Medical University of Lublin, ul. Chodźki 4A, 20-093 Lublin, Poland; ryszard.kocjan@umlub.pl

**Keywords:** star anise, *trans*-anethole, star anise oil, chemical composition, traditional applications, new directions of biological activity, cosmetic importance

## Abstract

*Illicium verum* Hook f. (star anise) is considered an important species in Traditional Chinese Medicine and is also used in contemporary medicine in East Asian countries. It occurs in natural habitats in southeastern parts of China and Vietnam, and is cultivated in various regions in China. The raw materials—*Anisi stellati fructus* and *Anisi stellati aetheroleum* obtained from this species exhibit expectorant and spasmolytic activities. *The European Pharmacopoeia* (4th edition) indicates that these raw materials have been used in allopathy since 2002. The biological activities of the above-mentioned raw materials are determined by the presence of valuable secondary metabolites such as monoterpenoids, sesquiterpenoids, phenylpropanoids, and flavonoids. Recent pharmacological studies on fruit extracts and the essential oil of this species have confirmed their antibacterial, antifungal, anti-inflammatory, and antioxidant activities and thus their medicinal and cosmetic value. The aim of this review was to examine the progress of phytochemical and pharmacological studies that focused on possible cosmetic applications. In addition to fruit extracts and essential oil, the current consensus on the safety of *trans*-anethole, which is the main compound of essential oil used in cosmetology, is underlined here.

## 1. Introduction

*Illicium verum* Hook f. (star anise, Chinese star anise) is a woody species commonly known as *ba jiao hui xiang* in China and is used in traditional Chinese medicine (TCM) as a therapeutic agent [1]. In line with the guidelines of the *Chinese Pharmacopoeia* [2], contemporary Chinese medicine recommends *I. verum* as a valuable medicinal plant. In addition, two raw materials obtained from *I. verum* (fruit—*Anisi stellati fructus* and essential oil—*Anisi stellati aetheroleum*) have been listed in *The European Pharmacopoeia* since 2002 (4th edition) [3]. Both of these materials exhibit expectorant and spasmolytic effects.

Nowadays, *I.* *verum* is an important medicinal plant worldwide. The recent scientific studies have proven that the fruit and essential oil of *I. verum* are characterized by biological activities such as antibacterial, antifungal, anti-inflammatory, and antioxidant effects [4]. The plant is also widely used in the food industry as a spice [1].

The main component of the *I. verum* essential oil is *trans*-anethole. It is extensively used in food, perfume, and pharmaceutical industries due to its sweet flavor and aromatic scent [5]. Moreover, according to recent studies, *trans*-anethole possesses antioxidant, anti-inflammatory and anti-obesity properties, which are also significant in terms of cosmetology and medicine.

This review presents the information collected from scientific reports on the biological properties of *I. verum* and *trans*-anethole and their potential medicinal and cosmetic applications.

## 2. General Characteristics

### 2.1. Botanical and Ecological Characteristics

**Figure 1:**
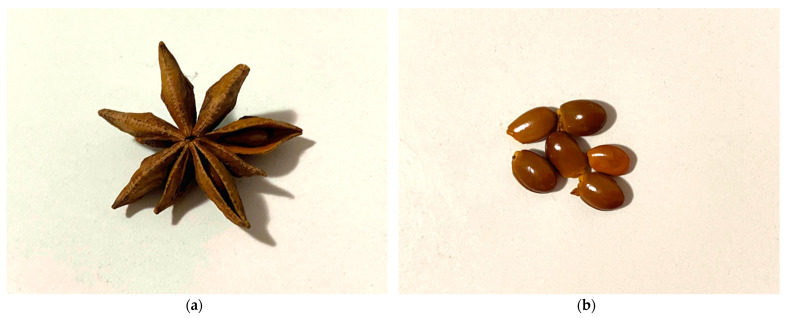
*I. verum:* (**a**) dried fruit; (**b**) seeds.

*Illicium verum* is an evergreen well-branched tree or shrub that measures around 8–15 m in height [4]. It was included in the Illiciaceae (Badianaceae) family in the older systematics. In the APG IV (2016) system, *I. verum* is classified under the genus *Illicium* belonging to the Schisandraceae family [6,7]. The bark of *I. verum* plants is white to light gray in color. The leaves are light green, lanceolate, leathery, and alternate, measure 6 to 12 cm long, and are located at the ends of branches [8,9,10]. The flowers are solitary, bisexual, white-yellow or greenish in color, and 1–7 cm in diameter. They grow either singly or arranged in clusters [8]. The fruit is star-shaped and has 6–10 capsule-like follicles with a small brown seed inside each (Figure 1). The seeds are ovoid with a shiny and smooth surface. Each part of the fruit carries an aromatic scent [8,10].

The species can be found in natural habitats in southeastern China and Vietnam. However, for commercial purposes, it is widely cultivated in China [1] as well as in Morocco, India, the Philippines, and some European countries, namely Spain, France, and Italy [11]. Its seeds were collected and brought to Europe from the Philippines for the first time by English sailor Thomas Cavendish in 1578 [8,11].

### 2.2. Chemical Characteristics

The major chemical compounds present in *I. verum* are phenylpropanoids, flavonoids, neolignans, monoterpenoids, and sesquiterpenoids.

The dominant component of the essential oil obtained from *I. verum* fruit is the phenylpropanoid compound *trans*-anethole. The average content of *trans*-anethole in the *I. verum* essential oil is around 72–92% [1,5,12]. 

The studies confirmed that the *trans*-anethole content in the essential oil is dependent on the applied extraction method. Wang et al. [13] analyzed the influence of three extraction techniques on the volatile contents: steam distillation (SD), solvent extraction (SE) and supercritical fluid extraction (SFE). The content of *trans*-anethole was as follows: 70.61% (SE), 77.31% (SFE) and 74.96% (SD). Additionally, the highest content of the other chemical compounds and the best quality of the essential oil was achieved after SFE. In another investigation by Sabry et al. [14], the content of *trans*-anethole obtained by the hydrodistillation (HD) method was smaller (47.16%) in comparison to the SFE used by Wang et al. [13]. 

Wei et al. [15] extracted the *I. verum* fruits with methyl alcohol (MA), ethyl acetate (EA) and petroleum ether (PE). As a result of this study, the yields of *trans*-anethole were not as effective and were equal to 7.5% (EA), 9.7% (MA) and 10.1% (PE).

**Figure 2:**
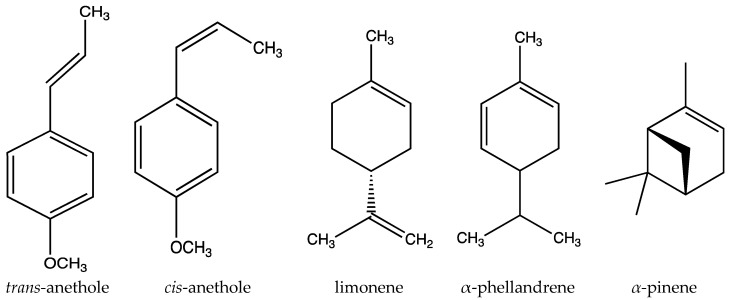
Chemical structures of selected compounds of *I. verum* essential oil.

Other compounds present in the essential oil of *I. verum* fruit are estragole (*p*-allyl anisole, methyl chavicol) (~2%), limonene (~2%), and *cis*-anethole (~0.5%) [16]. The essential oil also contains monoterpenoids (including α-pinene, *p*-cymene, eugenol, linalool, camphene, β-myrcene, *trans*-ocimene, terpinen-4-ol, α-terpineol, γ-terpineol, terpinolene, and γ-terpinene), and sesquiterpenoids (including *trans*-α-bergamotene, α-copaene, cubebene, cyperene, (+)-9-epiledene, β-elemene, α-phellandrene, foeniculin, α-caryophyllene, β-caryophyllene, and α-muurolene) [4,17]. In addition, *p*-anisaldehyde, 2-(1-cyclopentenyl)-furan, isobornyl thiocyanoacetate, and *trans*-chalcone have been detected (Table 1) [4,17,18]. The chemical structures of selected *I. verum* essential-oil compounds are shown in Figure 2.

*I. verum* essential oil is characterized by a licorice-like and sweet odor. The flavor of the essential oil is very similar to anise seed or fennel oil, but is stronger [11]. Hasegwa et al. [19] found that the key compound of *I. verum*, which determines its aroma, was *trans*-anethole, which has an aromatic herbaceous odor. The authors also claimed that the presence of methoxy and methyl groups within benzenoid rings also determines the characteristic star-anise-like aroma. Zhang et al. [20] also determined that the main aroma component of *I. verum* essential oil are *trans*-anethole, *p*-anisaldehyde, farnesol and estragole.

Besides essential oil, *I. verum* fruit contains flavonoids, such as kaempferol and quercetin and their glycosides, in minor amounts [1]. Furthermore, *I. verum* fruit also contains shikimic acid [1], fatty acids such as linoleic, stearic, and myristic acid [4], and alkylglucoside *R*-sec-butyl-d-glucopyranoside [1]. The two new, specific phenolic glucosides (*E*)-4-methoxy-2-(1′-propen-1′-yl)-phenol-1-*O*-α-*L*-arabinofuranosyl-(1‴→6″)-β-d-glucopyranoside and (*E*)-4-methoxy-2-(1′-propen-1′-yl)-phenol-1-*O*-α*-L*-rhamnopyranosyl- (1″→‘6′’)-β-d-glucopyranoside were isolated from the *I. verum* fruit [21].

The derivatives of *trans*-anethole, such as *threo*-anethole glycol and *erythro*-anethole glycol, were detected in the leaf extract of *I. verum* [1]. The leaves were also found to contain seco-cycloartane-3,4-seco-(24Z)-cycloart-4(28),24-diene-3,26-dioic acid, 26-methyl ester [22], and two biphenyl-type neolignans, namely verimol G and verimol H (Table 1) [23].

Sesquiterpene lactones and their derivatives (veranisatin A–C, tashironin, tashironin A, and 11-*O*-debenzoyl-11-*O*-2-methylcyclopent-1-enecarboxytashironin) have been isolated from the roots of *I. verum* [1,23]. In addition, phenylpropanoids such as illiverin A, 4-allyl-2-(3-methylbut-2-enyl)-1,6-methylenedioxybenzene-3-ol, illicinole, 3-hydroxy-4,5-methylenedioxyallyl-benzene, (−)-illicinone-A, and 4-allyl-4-(3-methylbut-2-enyl)-1,2-methylenedioxycyclohexa-2,6-dien-5-one were isolated from the ethanolic extract of the root of this species (Table 1) [22].

## 3. Ethnopharmacology and Potential Uses in Phytotherapy—General Information

The fruit of *I. verum* has been widely used in TCM and is recommended for the treatment of abdominal pain, lumbago, colic, and emesis [1]. The dried ripe fruits of *I. verum* have been described in the *Compedium of Materia Medica* since the 16th century [8].

According to the Chinese book *Herbal Essential Collections* (1505), *I. verum* fruits are recommended to treat cholera and fistula. In 1509, the *I. verum* ripe fruit (“bajiaohuixiang”, “daliao”) was mentioned in the book *Chinese Herbal Medicine* (“*Bencaopinhuijinyao*”) and can be used as a carminative. In the book *Herbal Positive*, *I. verum* is mentioned to eliminate teeth and mouse disease [8,24].

In the United States and Mexico, *I. verum* is used to eliminate colic pain in infants and stomach pain. The fruits also possess sedative activity and can treat nervousness and sleeplessness. It also forms part of the herbal mixtures used in gastrointestinal disorders in Cuba [1].

Since the 16th century, *I. verum* has been used as a fragrance and expectorant in Europe. *The European Pharmacopoeia* (4th edition, 2002) described two raw materials obtained from this species—*Anisi stellati fructus* (star anise fruit) and *Anisi stellati aetheroleum* (star anise oil)—which have similar properties to the essential oil obtained from *Pimpinella anisum* (Apiaceae). As the essential oil of *P. anisum* is very expensive, it can be replaced by *I. verum* essential oil, which is a cost-friendly alternative [25].

It should be noted that the fruit of *I. verum* cannot be easily distinguished from that of *Illicium anisatum* (Japanese star anise). Since *I. anisatum* seeds contain anisatin, shikimin, and shikimotoxin, they are highly toxic and can cause inflammation in the kidneys, urinary tract, and gastrointestinal tract [12,26].

## 4. Biological Activities Confirmed by Scientific Reports and Potential Applications in Cosmetology

### 4.1. Antibacterial Activity

Yang et al. [27] reported that the ethanolic extracts of *I. verum* fruits exhibited antibacterial activity against clinical drug-resistant isolates, namely *Acinetobacter baumannii*, *Pseudomonas aeruginosa*, and *S. aureus* bacteria with MIC (minimum inhibitory concentration) values of 0.15, 0.70, and 0.11 μg/mL, respectively.

Benmalek et al. [28] compared the antibacterial effects of the extracts of *I. verum*, *Crataegus oxyacantha* ssp. *monogyna*, and *Allium cepa* against two Gram-positive bacterial strains (*S. aureus* ATCC 25923 and *S. aureus* ATCC 43300) and two Gram-negative bacterial strains (*P. aeruginosa* ATCC27852 and *E. coli* ATCC 25992). The authors found that the extract of *I. verum* was the least effective and showed lower antibacterial activity compared to the extracts of the other two plants.

Luís et al. [16] demonstrated that the essential oil of *I. verum* showed antibacterial activity against *A. baumannii* LMG1025 and LMG1041, with MBC (minimum bactericidal concentration) values of 16 and 8 μg/mL, respectively.

Yang et al. [29] investigated the antibacterial activity of the *I. verum* extracts from leaves and twigs against nine antibiotic-resistant isolates including *Staphylococcus aureus, Pseudomonas aeruginosa* and *Acinetobacter baumannii.* It was revealed that the SFE (supercritical CO_2_ extraction) extracts both from the twigs and leaves exhibited broader antibacterial activity against all the tested strains than the TSE (traditional solvent extraction) extract. The DIZ (disk inhibitory zone) for the SFE extracts was equal to 9–22 mm. Moreover, the results of the disk-diffusion assay revealed that the SFE extracts exhibited stronger activity than TSE (MIC = 0.1–4.0 mg/mL; MBC = 0.2–4.5 mg/mL). In addition, the antibacterial activity was also evaluated within the main *I. verum* compounds: *trans*-anethole, anisyl aldehyde, anisyl acetone and anisyl alcohol. It was revealed that *trans*-anethole demonstrated antibacterial activity only against *A. baumannii* strains (MIC ≤ 0.1 mg/mL; MBC = 0.1 mg/mL). In the case of the remaining compounds (anisyl aldehyde, anisyl acetone, anisyl alcohol) the antibacterial activity was broader (MIC = 1.5–5.0 mg/mL; MBC = 2.5–6.5 mg/mL).

Sabry et al. [14] evaluated the antibacterial activities of *I. verum* volatile-oil extract and water extract against four bacterial strains: *Escherichia coli*, *Bacillus cereus*, *Salmonella typhi* and *S. aureus*. It was revealed that the volatile-oil extract showed stronger antibacterial activity than the water extract (MIC = 6.6–10.0 μL SAF/mL and MIC = 16.4–29.6 μL SAF/mL, respectively).

Li et al. [30] evaluated the antibacterial activity of *I. verum* essential oil, as well as anisic acid and shikimic acid, which occur in *I. verum* fruits. The antibacterial activity was evaluated against two Gram-positive bacteria: *S. aureus* (MRSA) and *S. pyogenes*, and three Gram-negative bacteria: *E. coli*, *S. typhi* and *P. aeruginosa*. It was found that anisic acid (MIC = 400–2000 μg/mL) and shikimic acid (MIC = 400–1600 μg/mL) were more effective than the *I. verum* essential oil (MIC = 1000–1600 μg/mL).

### 4.2. Antifungal Activity

Huang et al. [17] examined the antifungal activity of the essential oil obtained from *I. verum* fruit as well as its main component (*trans*-anethole) against eleven fungal species of plant pathogens (*Alternaria solani*, *Bipolaris maydis*, *Botryodiplodia theobromae*, *Fusarium graminearum*, *Fusarium oxysporum* f. sp. *cucumerinum*, *F. oxysporum* f. sp. *lycopersici*, *F. oxysporum* f. sp. *vasinfectum*, *Magnaporthe oryzae*, *Pythium aphanidermatum*, *Rhizoctonia cerealis*, and *Rhizoctonia solani*). The authors noted that both the essential oil and *trans*-anethole exhibited strong antifungal effects. Besides, they claimed that the antifungal activity of the essential oil can be attributed to the presence of *trans*-anethole.

Dzamic et al. [31] investigated the antifungal activity of *I. verum* essential oil against nineteen fungal species (human, plant and food pathogens), namely: *Alternaria alternata*, *Aspergillus niger*, *Aspergillus ochraceus*, *Aspergillus flavus*, *Aspergillus terreus*, *Aspergillus versicolor*, *Aureobasidium pullulans*, *Candida albicans, Cladosporium cladosporioides*, *Cladosporium fulvium*, *Fusarium tricinctum*, *Fusarium sporotrichioides*, *Mucor mucedo*, *Penicillium funiculosum*, *Penicillium ochrochloron*, *Phomopsis helianthi*, *Phoma macdonaldi*, *Trichoderma viride*, and *Trichophyton mentagrophytes.* Among these fungal species, the authors observed the strongest antifungal activity against *A. alternata*, *A. pullulans*, *C. cladosporioides, C. fulvium*, and *P. helianthi* (MIC = 2.5 µg/mL).

Yazdani et al. [32] tested the antifungal properties of the ethanolic extracts of *P. anisum* seed and *I. verum* fruit against five fungal species: *A. niger, C. albicans*, *Epidermophyton fluccosum*, *Microsporum canis*, and *T. mentagrophytes.* Their results revealed that the extract of *I. verum* fruit exhibited a stronger inhibitory activity against all the tested species (MIC = 4–16 mg/mL; minimal fungicidal concentration (MFC) = 8–256 mg/mL) compared to the extract of *P. anisum* seeds.

Aly et al. [33] analyzed the antifungal activity of *I. verum* essential oil against three fungal species: *A. flavus, Aspergillus parasiticus*, and *Fusarium moniliforme.* The authors noted that the essential oil inhibited the growth of all the tested species in a dose-dependent manner. The complete growth inhibition of *A. flavus* and *A. parasiticus* was observed at 200 ppm and of *F. moniliforme* at 400 ppm. Moreover, they investigated the antimycotoxigenic activity of *I. verum* essential oil against all the tested fungi strains and found that the percent of mycotoxin inhibition was dependent on the concentration of the essential oil, with 100% inhibition documented at 200 ppm.

Sabry et al. [14] evaluated the antifungal activities of *I. verum* volatile-oil extract and water extract against four fungal strains: *A. ochraceous*, *A. carbonarius*, *F.oxysporum*, *P. chrysogenum*. It was revealed that the volatile-oil extract showed a lower antifungal activity than the water extract (MFC= 133.8–178.8 SAF/mL and MFC = 52.6–73.4 μL SAF/mL, respectively).

### 4.3. Antioxidant Activity

Luís et al. [16] evaluated the antioxidant activity of *I. verum* essential oil using the 2,2-diphenyl-1-picrylhydrazyl (DPPH) free-radical-scavenging assay. The authors found that the essential oil exhibited a strong antioxidant activity (IC_50_ (minimum inhibitory concentration) = 3.46%), which was related to the high content of phenylpropanoids (92.2%) including *trans*-anethole. They assumed that the double bonds of *trans*-anethole and the synergistic effect of the different components of *I. verum* essential oil contributed to the observed antioxidant activity.

Dinesha et al. [34] analyzed the antioxidant activity of the aqueous extract of powdered *I. verum* fruit and noticed that at a concentration of 25 µg/mL the extract exhibited significant antioxidant activity against H_2_O_2_ and protected against DNA damage. The authors also investigated the antioxidant potential using the DPPH assay and reported that the antioxidant activity of the studied extract was associated with the high content of polyphenols.

Cheng-Hong et al. [35] tested the antioxidant activity of the ethanolic extracts of *I. verum* powdered fruits, which were fractioned by hexane, ethyl ether, chloroform, ethyl acetate, and supercritical CO_2_, by the DPPH test. They found that the ethyl-ether and ethyl-acetate fractions showed the highest antioxidant potential (IC_50_ = 57.43 and 38.60 ppm, respectively). In addition, the ethyl-acetate fraction had the highest total phenolic and total flavonoid content, which can be linked to the confirmed antioxidant effect of the extracts.

Li et al. [30] evaluated the antioxidant activity of *I. verum* essential oil, as well as anisic acid and shikimic acid. The antioxidant activity evaluated with the DPPH assay showed that star anise oil, anisic acid and shikimic acid exhibited slight antioxidant activity compared to butylated hydroxytoluene (BHT) (IC_50_ = 1.32 mg/mL), with an IC_50_ equal to 9.88, 8.04 and 8.96 mg/mL, respectively.

### 4.4. Anti-Inflammatory Activity

Sung et al. [36] confirmed the anti-inflammatory activity of the ethanolic extract of *I. verum* fruits in a human keratinocyte cell line (HaCaT). Their study revealed that the extract suppressed the mRNA expression of pro-inflammatory cytokines IL-6 and IL-1β induced by TNF-α/IFN-γ. Furthermore, the extract regulated the activation of the TARC/CCL17 and MDC/CCL22 chemokines. Additionally, the translocation of the nuclear factor NF-κB into the cell nucleus, phosphorylation, and iκBα degradation were inhibited. *Trans*-anethole isolated from the *I. verum* extract (~2.14%) also showed anti-inflammatory activity by reducing the protein expression of TARC, MDC, and cytokines IL-6 and IL-1β.

## 5. Uses Based on the CosIng Database

According to the CosIng (Cosmetic Ingredient) database elaborated by the European Commission [37], *I. verum* can be used for the production of cosmetics. The following raw materials of *I. verum* can be used for this purpose: fruit extract, fruit essential oil, fruit hydrolat, fruit powder, and seed or leaf essential oil (Table 2 and Table 3). The extract of *I. verum* fruit can be used as a skin conditioning component and to mask unpleasant odors. The essential oil obtained from its seeds can be used as a fragrance or an oral-hygiene ingredient. Similarly, the essential oil obtained from *I. verum* leaves can be used as a skin-conditioning agent or as a fragrance and also has deodorizing properties. Fruit hydrolat can also be used as a fragrance [37].

## 6. Safety of Use

According to the Flavor and Extract Manufacturers Association (FEMA) [38], the main component of *I. verum* essential oil, *trans*-anethole, is “generally recognized as safe” (GRAS).

A report of the European Medicines Agency (EMA—The European Agency for the Evaluation of Medical Products and Veterinary Medicines and Information Technology Unit) [39] stated that *I. verum* fruit and essential oil can be used as an expectorant or stomachic in humans. In addition, it can be used as a spice and in alcoholic beverages, sweets, or toothpastes. The recommended average daily dose of *I. verum* fruit for humans is 3 g and that of essential oil is 0.3 g. The same report also indicated that *I. verum* fruit can be included as a component in veterinary preparations at a concentration of 2.88%, along with other active ingredients. The common uses of preparations containing *I. verum* in cattle, sheep, and goats are to treat gastric disorders such as forestomach atony or acute indigestion. For cattle weighing more than 200 kg, the appropriate dose of *I. verum* is 20 g, while for sheep and goats it is 15 g lower [39].

Despite its safety, in 2003 the U.S. Food and Drug Administration (FDA) issued a warning against the consumption of teas containing *I. verum* fruit, which was linked with side effects such as vomiting, nausea, convulsions, hypertonia, hypothermia and rapid eye movements. It was also reported that the teas can be contaminated with toxic *I. anisatum* [40]. 

Nakamura et al. [41] reported that the oral administration of veranisatin A, veranisatin B and veranisatin C caused acute toxicity in animal studies.

## 7. *Trans*-Anethole as the Main Active Component of *I. verum Essential Oil* —Chemical Characteristics, Importance in Cosmetology, and Safety of Use

### 7.1. General Characteristics

*Trans*-anethole (structurally 1-methoxy-4-[1(*E*)-propenyl] benzene) is an isomer of anethole (*E*-anethole). It is the dominant component of the essential oil obtained from *I. verum* fruit and can also be obtained from the seeds of *P. anisum* and *Foeniculum vulgare.*

*Trans*-anethole is volatile and slightly soluble in water, but well soluble in ethanol. It is characterized by a sweet and herbal fragrance, and thus used in perfumery and the food, cosmetic, and pharmaceutical industries [5,42]. The CosIng database indicates that anethole (without specifying its isomeric forms) can be used as a fragrance or denaturant [37].

### 7.2. Biological Activity and Potential Cosmetological Applications

#### 7.2.1. Antibacterial Activity

De et al. [43] proved that *trans*-anethole obtained from *I. verum* fruit exhibited a significant antimicrobial effect, with the best activity observed against *S. lutea* (MIC = 5 mg/mL), *Bacillus subtilis* (MIC = 5 mg/mL), *Bacillus megaterium* (MIC = 5 mg/mL), and *R. leguminosarum* (MIC = 5 mg/mL).

Kwiatkowski et al. [44] reported that *trans*-anethole at a concentration of 4% exhibited antibacterial activity against *S. aureus*. Moreover, their study revealed that *trans*-anethole enhanced the effectiveness of mupirocin (MUP) when used in combination and can therefore be included in MUP-based preparations. The MIC of MUP combined with *trans*-anethole was <0.064 µg/mL for *S. aureus* strains (86%).

Hancęr et al. [45] studied the impact of *trans*-anethole on quorum sensing (QS) and showed that *trans*-anethole displayed inhibitory activity against QS as was observed by a blue ring around *Escherichia coli* QSIS1. In addition, *trans*-anethole used at a concentration of 6 mM decreased the expression of *lasB* by about 57% (Table 4).

#### 7.2.2. Antifungal Activity

Huang et al. [17] found that *trans*-anethole exerted a significant antifungal effect on 11 fungi strains (plant pathogens): *Alternaria solani*, *Bipolaris maydis*, *Botryodiplodia theobromae*, *Fusarium graminearum*, *F. oxysporum* f. sp. *cucumerinum*, *F. oxysporum* f. sp. *lycopersici*, *F. oxysporum* f. sp. *vasinfectum*, *Magnaporthe oryzae*, *Pythium aphanidermatum*, *Rhizoctonia cerealis*, and *R. solani* (IC_50_ = 0.06–0.25 mg/mL) (Table 4).

#### 7.2.3. Antioxidant Activity

Luís et al. [16] evaluated the antioxidant activity of *I. verum* fruit using the DPPH assay and found that its essential oil possessed antioxidant properties, which was assumed to be associated with the presence of *trans*-anethole, and particularly with its double bonds in the molecules (Table 4).

#### 7.2.4. Anti-Inflammatory Activity

Kim et al. [46] investigated the anti-inflammatory effect of *trans*-anethole in a mouse model of chronic obstructive pulmonary disease induced by porcine pancreatic elastase (PPE) and lipopolysaccharide (LPS). *Trans*-anethole was orally administered to mice at four doses (62.5, 125, 250, and 500 mg/kg of body weight). After 2 h of *trans*-anethole administration, the mice were treated with PPE and LPS. The results revealed that *trans*-anethole, similar to glucocorticoid dexamethasone, decreased the activity of lactate dehydrogenase. Additionally, it decreased the expression of proinflammatory cytokines IL-6 and TNF-α, and reduced blood pressure.

Sung et al. [47] studied the anti-inflammatory activity of *I. verum* extract and *trans*-anethole in mice with ovalbumin-induced asthma. *Trans*-anethole was orally administered at two doses (2 and 20 mg/kg of body weight) within 4 weeks. The results revealed that *trans*-anethole decreased inflammation in the airways, which was evidenced by reduced inflammatory cell infiltrates and fibrosis. Moreover, an increased Fox3 expression was noted. *Trans*-anethole also reduced IL-4 expression in the supernatant of splenocyte cultures and increased IFN-γ expression.

Sung et al. [36] also investigated the anti-inflammatory effect of *I. verum* fruit extract and its main compound *trans*-anethole in the human keratinocyte HaCaT cell line. The authors observed that *trans*-anethole exhibited anti-inflammatory activity in the studied cells, which was evidenced by the reduced protein expression of TARS, MDC, IL-4, and IL-1β without any accompanying cytotoxic effect.

Moradi et al. [48] studied the anti-inflammatory effect of *trans*-anethole in rats with periodontitis (PD) induced by the administration of 30 μg of *E. coli* LPS for 10 days. *Trans*-anethole was intraperitoneally (i.p.) administered at two doses (10 and 50 mg/kg), before 20 min of LPS injection. The results showed that, compared to ketoprofen-treated mice (10 mg/kg, i.p.), the mice treated with *trans*-anethole showed a significantly higher anti-inflammatory effect, as could be observed by a decrease in IL-1β and TNF-α expression (Table 4).

#### 7.2.5. Activity against Obesity

Kang et al. [49] investigated the effect of *trans*-anethole on high-fat-diet-induced obesity in mice. The authors found that *trans*-anethole increased mitochondrial biogenesis in white adipocytes, which was confirmed by the increased expression of COX4, Nrf1, MtDNA, and Tfam. Additionally, *trans*-anethole induced browning in white adipocytes by stimulating the expression of β3-AR and brown-adipose-tissue proteins (Ppargc1α, Prdm16, UCP1, PKA). *Trans*-anethole also induced SIRT1 expression, and as a result, increased the expression of adipose-tissue-browning markers (UCP1, pAMPK, PRDM16, PGC-1α). Furthermore, *trans*-anethole increased adipogenesis, lipogenesis, and lipolysis, and induced the expression of beige adipocyte genes (Ucp1, Cd137, Cited 1, Tbx1, Tmem26) (Table 4).

### 7.3. Safety of Use

In 1965, the FEMA [38] approved *trans*-anethole as a safe compound and recognized it with the GRAS status. In 1979 and 1997, the GRAS status of *trans*-anethole was reaffirmed [38].

According to the EFSA [50], *trans*-anethole is a safe compound that can be used as a flavoring agent. However, it has warned that *trans*-anethole can cause skin sensitization when applied topically [50]. 

The European Chemical Agency (ECHA) [51] classified *trans*-anethole under Category 1 as a skin sensitizer in the Global Harmonized System of Classification and Labeling of Chemicals (GHS).

When exposed to light and high temperature, *trans*-anethole converts to *cis*-anethole (*Z*-anethole), which is characterized by very high toxicity. Nevertheless, *I. verum* essential oil has a negligible amount of *cis*-anethole (approximately 0.1–1.7%) and hence is considered as a safe raw material [5,42].

Akçan et al. [42] reported that *trans*-anethole is unlikely to cause genotoxicity. *Trans*-anethole can potentially exhibit toxicity in a dose-dependent manner, which is probably related to the metabolite - anethole epoxide.

Besides its safety, it was also reported that anethole can be a skin sensitizer. Poon et al. [52] investigated that *trans*-anethole (2% in petrolatum), which was the main flavoring component of the toothpaste, caused a contact allergy of a 65-year-old woman. In another investigation, 100 patients were tested with three star-anise-oil concentrations (0.5%, 1%, 2%) [53]. It was proved that star-anise oil caused skin sensitization in 1–2% concentrations within 5% of tested patients. Garcia-Bravo et al. [54] reported that *trans*-anethole had an influence on the development of dermatitis in two bakers cooking cakes with a star anise oil as a flavouring agent. The positive reaction was observed to star anise oil and its main component anethole.

## 8. Conclusions

*Illicium verum* is an important species that was widely used in TCM. Both the fruit and the essential oil are pharmacopoeial raw materials and have been used for many years to treat rheumatism, insomnia, or digestive disorders. The valuable properties of *I. verum* fruits are attributed to its unique chemical composition with abundant amounts of phenylpropanoids, mono- and sesquiterpenoids.

Numerous scientific studies have shown that *I. verum* fruit and essential oil exhibit strong biological activities such as antibacterial, antifungal, anti-inflammatory, and antioxidant effects. Due to these properties, *I. verum* can be used in the cosmetic industry.

The essential oil of *I. verum* is a rich source of *trans*-anethole (over 72%). *Trans*-anethole (*E*-anethole) is an isomer of anethole and gives *I. verum* its characteristic aroma, which favors the application of this species in perfumery and cosmetic industry.

Numerous investigations confirm that *I. verum* can be effective as an antibacterial or antifungal agent in food manufacturing or medicne. It can be used in the treatment of a dry cough or bronchitis. The anti-inflammatory activity of *I. verum* enables the potential use of the plant in cases of skin diseases. Due to *I. verum* having proven expectorant properties, it can also be widely used for the production of antitussives. The pleasant anise-like smell can be used in the production of natural breath fresheners.

Besides medical applications, *I. verum* can offer a wealth of opportunities for cosmetics applications, which are largely determined by the presence of *trans*-anethole. According to its antioxidant activities, it can be extensively used not only in perfume production, but also in anti-aging cosmetics. Besides, proven anti-obesity properties make the *trans*-anethole a potential natural dietary supplement.

## Figures and Tables

**Table 1:** Chemical composition of *I. verum*.

Group of Compounds	Raw Material	Compound Name	References
Phenolic compounds	Essential oil	*Trans*-anethole, *cis*-anethole, estragole	[16]
	Fruit	Shikimic acid	[4]
	Root	Illiverin A, 4-allyl-2-(3-methylbut-2-enyl)-1,6-methylenedioxybenzene-3-ol, illicinole, 3-hydroxy-4,5-me-thylenedioxyallyl-benzene, (−)-illicinone-A, 4-allyl-4-(3-methylbut-2-enyl)-1,2-methylenedioxycyclohexa-2,6-dien-5-one, 3,4-seco-(24Z)-cycloart-4(28),24-diene-3,26-dioic acid, 26-methyl ester	[22]
Monoterpenoids	Essential oil	α-Pinene, p-cymene, limonene, linalool, terpinen-4-ol, α-terpineol, eugenol, γ -terpineol, ơ-3-carene, camphene, β-myrcene, *trans*-ocymene, terpinolene, γ -terpinene	[4,17]
Sesquiterpenoids	Essential oil	α-Phellandrene, α-muurolene, β-caryophyllene, α-copaene, *trans*-α-bergamotene, foeniculin, β-elemene, cyperene, α-caryophyllene, (+)-9-epiledene, cubebene	[4,17]
	Root	Tashironin, tashironin A, 11-*O*-debenzoyl-11α-*O*-2-methylcyclopent-1-enecarboxyltashironin, veranisatins A–C	[1,23]
Flavonoids	Essential oil	*Trans*-chalcone	[18]
	Fruit	Kaempferol and glucosides, quercetin and glucosides	[1]
Fatty acids	Fruit	Linoleic acid, stearic acid, myristic acid	[4]
Alkylglucosides	Fruit	R-sec-butyl-d-glucopyranoside	[4]
Biphenyl-type neolignans	Leaf	Verimol G and verimol H, 4,4′-dihydroxy-3,3′-dimethoxy-9,9′-epoxylignan	[23]
Aldehydes	Essential oil	*p*-Anisaldehyde	[4,18]
Other	Essential oil	Anisoxide, 2-(1-cyclopentenyl)-furan, isobornyl thiocyanoacetate	[8,17]

**Table 2:** Uses of *I. verum* in cosmetics according to CosIng database.

Form	Function
*Illicium verum fruit extract*	Perfuming, skin conditioning
*Illicium verum fruit water*	Fragrance, perfuming
*Illicium verum fruit powder*	Exfoliating
*Illicium verum fruit oil*	Perfuming
*Illicium verum seed oil*	Fragrance, oral care, tonic
*Illicium verum leaf oil*	Flavoring, fragrance, skin conditioning

**Table 3:** *I. verum* as a cosmetic ingredient.

Manufacturer	Country	Trade Name	Form	Form of *I. verum* in a Composition of the Cosmetic (INCI) According to the Manufacturer	Function
MUGLER www.mugler.com	France	Cuir Impertinent	Perfumed water	Star Anise—top note	Perfuming
Tonymoly www.tonymoly.us	Korea	I’M POMEGRANATE Mask Sheet	Mask sheet	*Illicium verum* (Anise) Fruit Extract	Moisturizing, elasticizing
		I’M REAL Makgeolli Mask Sheet	Mask sheet	*Illicium verum* (Anise) Extract	Smoothing, moisturizing
		I’M AVOCADO Nutrition Beauty Mask Sheet	Mask sheet	*Illicium verum* (Anise) Fruit Extract	Nourishing, revitalizing
		I’M RED WINE Pore Care Beauty Mask Sheet	Mask sheet	*Illicium verum* (Anise) Fruit Extract	Cleansing, tightening
Elizavecca www.elizavecca.com	Korea	Pore Clean Up AHA Fruit Toner	Cleansing toner	*Illicium verum* (Anise) Fruit Extract	Exfoliating, cleansing, moisturizing
		Gold CF-NEST Collagen Jella Pack Beauty Mask	Face mask	*Illicium verum* (Anise) Fruit Extract	Elasticizing, firming, exfoliating
		Gold CF-NEST, White Bomb Eye Cream	Eye cream	*Illicium verum* (Anise) Fruit Extract	Brightening, smoothing
		Skin Liar Primer	Face primer	*Illicium verum* (Anise) Fruit Extract	Brightening, smoothing
		Perfect Sparking Peeling Pad	Peeling pad	*Illicium verum* (Anise) Fruit Extract	Cleansing, exfoliating
Klairs www.klairscosmetics.com	Korea	Rich Moist Soothing Serum	Serum	*Illicium verum* (Anise) Fruit Extract	Moisturizing, soothing
		Freshly Juiced Vitamin Drop Serum	Serum	*Illicium verum* (Anise) Fruit Extract	Brightening, smoothing, improving the skin condition
		Rich Moist Foaming Cleanser	Foaming cleanser	*Illicium verum* (Anise) Fruit Extract	Moisturizing, soothing, refreshing, cleansing
		Supple Preparation All Over Lotion	Lotion	*Illicium verum* (Anise) Fruit Extract	Moisturizing, protective, soothing
Son & Park www.en.sonandpark.com	Korea	Beauty Water	Cleansing water/ toner	*Illicium verum* (Anise) Fruit/Seed Oil	Moisturizing, refreshing, exfoliating, cleansing
COSRX www.cosrx.com	Korea	Low pH Barrier Mist	Face mist	*Illicium verum* (Anise) Fruit Extract	Moisturizing, refreshing, elasticizing, restoring pH balance
D’Alba www.dalbaglobal.com	Korea	White Truffle Whitening	Cream	*Illicium verum* (Anise) Fruit Extract	Brightening, protective, elasticizing
Missha www.missha.com	Korea	Real Solution Tencel Sheet Mask	Sheet mask	*Illicium verum* (Anise) Fruit Extract	Moisturizing, soothing, strengthening the skin natural barrier
Aromatica www.thearomatica.com	Korea	Rosemary Scalp Scaling Shampoo	Shampoo	*Illicium verum* (Anise) Fruit Extract	Nourishing, exfoliating
Doctor Babor www.babor.com	Germany	3D Hydro Gel Face Mask	Face mask	*Illicium verum* (Anise) Fruit Extract	Moisturizing, elasticizing, refreshing, toning
Oceanic www.oceanic.com.pl	Poland	Facial Sheet Mask Rose + Phytocollagen	Sheet mask	*Illicium verum* (Anise) Fruit Extract	Regenerating, smoothing, improving the skin condition
		Facial Sheet Mask Lemon + Vitamin C	Sheet mask	*Illicium verum* (Anise) Fruit Extract	Brightening, smoothing, rejuvenating, revitalizing
EO Laboratorie www.ec-l.ru/en	Russia	Smoothness& Tonus Scrub	Scrub	*Illicium verum* Oil	Elasticizing, moisturizing, softening, exfoliating
PIXI www.pixibeauty.com	United States	Rose Glow Mist	Mist	*Illicium verum* (Anise) Fruit Extract	Moisturizing, refreshing, elasticizing, protective against free radicals
		Glow Glycolic Boost	Sheet mask	*Illicium verum* (Anise) Fruit Extract	Brightening, moisturizing
		Rose Caviar Essense	Flower oil	*Illicium verum* (Anise) Fruit Extract	Moisturizing, softening, nourishing
Eco-Dent www.eco-dent.com	Unites States	GentleFloss Mint	Dental floss	*Illicium verum* Oil	Refreshing, anti-cavity
Jason Natural www.jason-personalcare.com	United States	Powersmile, Antiplaque &Whitening Toothpaste	Toothpaste	*Illicium verum* (Anise) Fruit/Seed Oil	Whitening, reducing unpleasant odor
Kerosene www.houseofkerosene.com	Unites States	Black Vines	Perfumed water	Star Anise	Perfuming
Dr Bronner’s www.drbronner.com	Great Britain	Peppermint Toothpaste	Toothpaste	Organic *Illicium verum* (Anise) Seed Oil	Whitening, refreshing, reducing plaque
Jo Malone www.jomalone.com	Great Britain	Vanilla & Anise Cologne	Cologne	Star Anise—top note	Perfuming
DIESEL www.diesel.com	Italy	Loverdose	Perfumed water	Star Anise—top note	Perfuming
OUDFACTORY www.oudfactory.com	United Arab Emirates	Moya Kvitka	Perfumed water	Star Anise	Perfuming

**Table 4:** Biological activity of *I. verum* and *trans*-anethole with potential applications in cosmetology.

Biological Activity	Characteristics	Tested Raw Material/Chemical Compound	References
Antibacterial activity	Inhibitory activity against *Staphylococcus aureus*	*Trans*-anethole	[44]
	Inhibitory activity against: *Escherichia coli quorum sensing* capacity, lasB expression, and *Pseudomonas aeruginosa* PAO1 virulence factor production	*Trans*-anethole	[45]
	Inhibitory activity against: *Acinetobacter baumannii, Pseudomonas aeruginosa, Staphylococcus aureus*	Ethanol extract of the *I. verum* herb	[27]
	Inhibitory activity against Gram-positive bacteria: *Bacillus subtilis, B. cereus*, *B. licheniformis, B. megatarium*, *Sarcina lutea, Staphylococcus aureus* Inhibitory activity against Gram-negative bacteria: *Agrobacterium tumefacienes, Bradyrhizobium japonicum, Escherichia coli*, *Klebsiella pneumoniae*, *K. aerogenes*, *Rhizobium leguminosarum*	Extract from *I. verum* fruit	[43]
	Inhibitory activity against: *Bacillus megatarium, B. subtilis, Rhizobium leguminosarum, Sarcina lutea.*	*Trans*-anetole	[43]
	Inhibitory activity against: *Acinetobacter baumannii*	Essential oil of *I. verum*	[16]
Antifungal activity	Inhibitory activity against: *Alternaria solani*, *Bipolaris maydis*, *Botryodiplodia theobromae*, *Fusarium graminearum*, *F. oxysporum* f. sp. *cucumerinum*, *F. oxysporum* f. sp. *lycopersici*, *F. oxysporum* f. sp. *vasinfectum*, *Magnaporthe oryzae*, *Pythium aphanidermatum*, *Rhizoctonia cerealis* and *R. solani.*	Essential oil from *I. verum* fruit, and isolated *trans*-anethole	[17]
	Inhibitory activity against: *Alternaria alternata*, *Aspergillus niger*, *A. ochraceus*, *Aspergillus flavus*, *A. terreus*, *A. versicolor*, *Aureobasidium pullulans*, *Candida albicans, Cladosporium cladosporioides*, *C. fulvium*, *Fusarium tricinctum*, *F. sporotrichioides*, *Mucor mucedo*, *Penicillium funiculosum*, *P. ochrochloron*, *Phomopsis helianthi*, *Phoma magdonaldii*, *Trichoderma viride*, *Trichophyton mentagrophytes*	Essential oil of *I. verum* fruit	[31]
	Inhibitory activity against: *Aspergillus niger, Candida albicans*, *Epidermophyton floccosum*, *Microsporum canis* and *Trichophyton mentagrophytes*	Ethanol extract of the *I. verum* fruit	[32]
	Inhibitory activity against aflatoxin B1 and fumonisin B1, 100% antifungal activity in a dose dependent manner (200 ppm)	Essential oil of *I. verum* fruit	[33]
Antioxidant activity	Strong antioxidant activity in DPPH test (IC50 = 3.46%)	Essential oil of *I. verum*	[16]
	Protective activity against DNA damage caused by hydrogen peroxide, inhibitory activity against human peripheral lymphocyte cell death, lipid peroxide inhibitory activity and hydroxyl radicals	Aqueous extract of the *I. verum* fruit	[34]
	Strong antioxidant activity in DPPH test	Ethyl acetate fraction from *I. verum* fruit	[35]
Anti-inflammatory activity	Inhibitory activity against mRNA expression induced by TNF-a /IFN-γ and protein expression of thymus, regulation of chemokine activation (TARC/CCL17), macrophage-derived chemokine (MDC/CCL22) oral interleukin (IL-6 i IL-1β)	Ethanol extract of the *I. verum* fruit	[36]
	Inhibitory activity against nuclear factor (NF-κB) translocation into the nucleus, phosphorylation and IκBα degradation	*Trans*-anethole isolated from *I. verum* fruit	[36]
	Decreased activity of lactate dehydrogenase, blood pressure regulation, the reduction of level of pro-inflammatory cytokines (IL-4, TNF-α)	*Trans*-anethole	[46]
	Airway hyperresponsiveness suppression, inhibitory activity against immunoglobulin E (IgE) production, reduced production of interleukin 4 (IL-4) in the supernatant of splenocyte cultures	*Trans*-anethole	[47]
	Inhibitory activity against IL-1β i TNF-α expression	*Trans*-anethole	[48]
Anti-obesity activity	Adipocytes browning induction, lipolysis activation, inhibitory activity against adipogenesis and lipogenesis	*Trans*-anethole	[49]

## Data Availability

Not applicable.
